# Crystal structure of 4-{(*E*)-[2-(pyridin-4-ylcarbon­yl)hydrazin-1-yl­idene]meth­yl}phenyl acetate monohydrate

**DOI:** 10.1107/S2056989014027819

**Published:** 2015-01-03

**Authors:** Riya Datta, V. Ramya, M. Sithambaresan, M. R. Prathapachandra Kurup

**Affiliations:** a Department of Chemistry, Christ University, Hosur Road, Bangalore 560 029, India; b Department of Chemistry, Faculty of Science, Eastern University, Sri Lanka, Chenkalady, Sri Lanka; c Department of Applied Chemistry, Cochin University of Science and Technology, Kochi 682 022, India

**Keywords:** crystal structure, hydrazone, aroyl hydrazone, hydrogen bonding

## Abstract

The asymmetric unit of the title compound, C_15_H_13_N_3_O_3_·H_2_O, comprises a 4-{(*E*)-[2-(pyridin-4-ylcarbon­yl)hydrazinyl­idene]meth­yl}phenyl acetate mol­ecule and a solvent water mol­ecule linked by O—H⋯O and O—H⋯N hydrogen bonds from the water mol­ecule and a C—H⋯O contact from the organic mol­ecule. The compound adopts an *E* conformation with respect to the azomethine bond and the dihedral angle between the pyridine and benzene rings is 21.90 (7)°. The azomethine bond [1.275 (2) Å] distance is very close to the formal C=N bond length, which confirms the azomethine bond formation. An extensive set of O—H⋯O, O—H⋯N, N—H⋯O and C—H⋯O hydrogen bonds builds a two-dimensional network progressing along the *c* axis.

## Related literature   

For biological applications of hydrazone derivatives, see: Sreeja *et al.* (2004 ▸); Prasanna & Kumar (2013 ▸). For the synthesis of related compounds, see: Joseph *et al.* (2013 ▸); Thilagavathi *et al.* (2010 ▸). For the anti­cancer activity of hydrazones against cervical cancer, see: Nair *et al.* (2014 ▸).
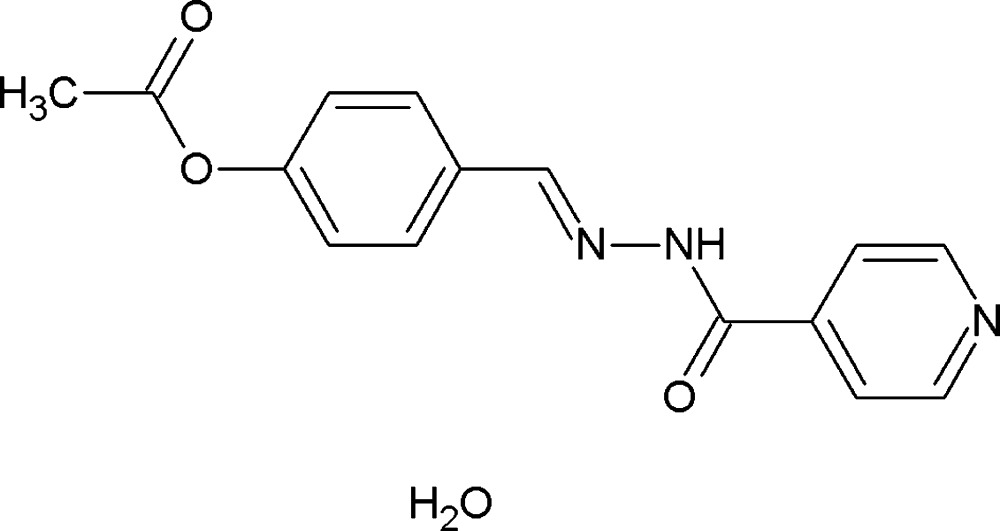


## Experimental   

### Crystal data  

C_15_H_13_N_3_O_3_·H_2_O
*M* *_r_* = 301.30
Monoclinic, 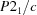

*a* = 17.3297 (15) Å
*b* = 7.3058 (7) Å
*c* = 12.4632 (10) Å
β = 111.034 (3)°
*V* = 1472.8 (2) Å^3^
*Z* = 4
Mo *K*α radiation
μ = 0.10 mm^−1^
*T* = 296 K
0.50 × 0.45 × 0.40 mm

### Data collection  

Bruker APEXII CCD diffractometer
Absorption correction: multi-scan (*SADABS*; Bruker, 2004 ▸) *T*_min_ = 0.951, *T*_max_ = 0.961
8648 measured reflections
2614 independent reflections
2153 reflections with *I* > 2σ(*I*)
*R*_int_ = 0.028

### Refinement  

*R*[*F* ^2^ > 2σ(*F* ^2^)] = 0.037
*wR*(*F* ^2^) = 0.113
*S* = 0.94
2614 reflections
213 parameters
4 restraints
H atoms treated by a mixture of independent and constrained refinement
Δρ_max_ = 0.21 e Å^−3^
Δρ_min_ = −0.17 e Å^−3^

### 

Data collection: *APEX2* (Bruker, 2004 ▸); cell refinement: *APEX2* and *SAINT* (Bruker, 2004 ▸); data reduction: *SAINT* and *XPREP* (Bruker, 2004 ▸); program(s) used to solve structure: *SHELXS97* (Sheldrick, 2008 ▸); program(s) used to refine structure: *SHELXL2014* (Sheldrick, 2008 ▸); molecular graphics: *ORTEP-3 for Windows* (Farrugia, 2012 ▸) and *DIAMOND* (Brandenburg, 2010 ▸); software used to prepare material for publication: *SHELXL97* and *publCIF* (Westrip, 2010 ▸).

## Supplementary Material

Crystal structure: contains datablock(s) I, global. DOI: 10.1107/S2056989014027819/sj5434sup1.cif

Structure factors: contains datablock(s) I. DOI: 10.1107/S2056989014027819/sj5434Isup2.hkl

Supporting information file. DOI: 10.1107/S2056989014027819/sj5434Isup3.cml

**Fig. 1.**
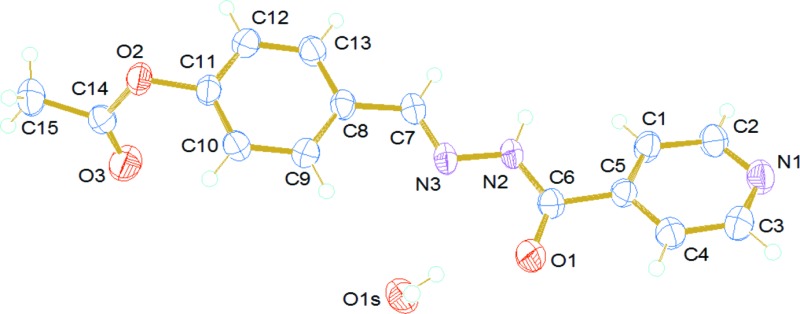
An ORTEP view of the compound, with 50% probability displacement ellipsoids for the non-H atoms.

ORTEP . DOI: 10.1107/S2056989014027819/sj5434fig1.tif
An *ORTEP* view of the compound, with 50% probability displacement ellipsoids for the non-H atoms.

**Fig. 2.**
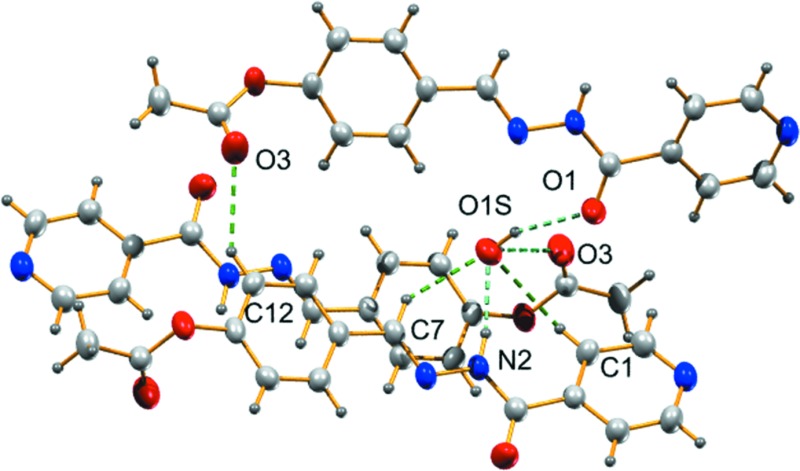
Graphical representation showing hydrogen bonding interactions in the crystal structure of [C15H13N3O3]·(H2O).

**Fig. 3.**
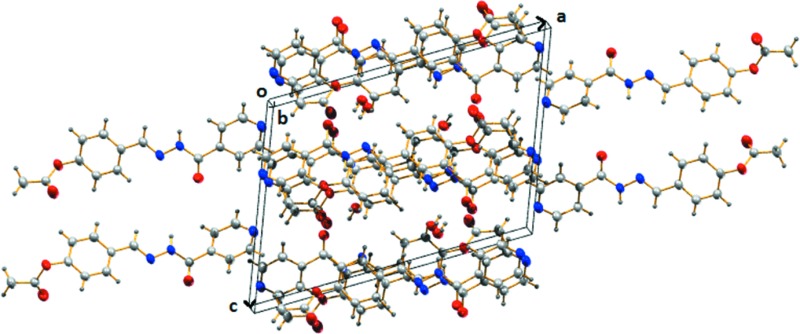
The hydrogen bonding interactions build a double layer progressing along the c axis in the title compound.

**Fig. 4.**
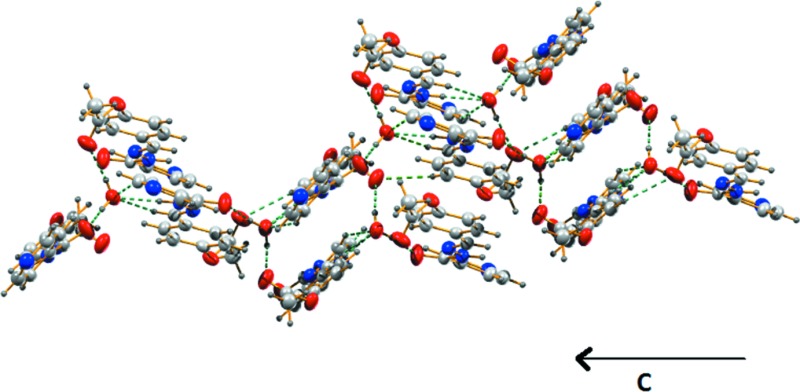
A view of the overall crystal packing along the a axis.

15 13 3 3 2 . DOI: 10.1107/S2056989014027819/sj5434fig2.tif
Graphical representation showing hydrogen bonding inter­actions in the crystal structure of [C_15_H_13_N_3_O_3_]·(H_2_O).

c . DOI: 10.1107/S2056989014027819/sj5434fig3.tif
The hydrogen bonding inter­actions build a double layer progressing along the *c* axis in the title compound.

a . DOI: 10.1107/S2056989014027819/sj5434fig4.tif
A view of the overall crystal packing along the *a* axis.

CCDC reference: 1040455

Additional supporting information:  crystallographic information; 3D view; checkCIF report

## Figures and Tables

**Table 1:** Hydrogen-bond geometry (, )

*D*H*A*	*D*H	H*A*	*D* *A*	*D*H*A*
C1H1O1*S* ^i^	0.93	2.56	3.375(2)	147
C7H7O1*S* ^i^	0.93	2.56	3.3655(19)	145
C12H12O3^i^	0.93	2.54	3.329(2)	143
N2H2O1*S* ^i^	0.88(1)	2.08(1)	2.9529(18)	170(2)
O1*S*H1*S*N3	0.86(2)	2.65(2)	3.2897(18)	133(2)
O1*S*H1*S*O1	0.86(2)	2.02(2)	2.8382(17)	159(2)
O1*S*H2*S*O3^ii^	0.86(2)	2.38(2)	3.1754(19)	154(2)

Symmetry codes: (i) 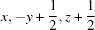; (ii) 
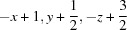
.

## supplementary crystallographic information

### S1. Structural commentary

Hydrazone derivatives are found to have structural diversity due to their coordinative ability (Sreeja *et al.*, 2004; Prasanna & Kumar, 2013) arising from thio­amido-thio­iminol tautomerism. Ruthenium(II) complexes with such compounds as ligands have been shown to function as catalysts (Thilagavathi *et al.*, 2010). The title compound [C_15_H_13_N_3_O_3_].(H_2_O) adopts an *E* configuration with respect to C7═N3 bond and O1 and N3 are cis with respect to the C6—N2 bond (Fig. 1). The dihedral angle between the pyridine and benzene rings is 21.90 (7) Å. The C7═N3 [1.275 (2) Å] bond distances are very close to the formal C═N bond length, which confirms the azomethine bond formation.

There are four classical inter­molecular O—H···O, O—H···N and N—H···O hydrogen bonds and three non-classical C—H···O inter­actions, (Table 1, Figure 2). These inter­molecular hydrogen bonds build a two-dimensional network progressing along the *c* axis (Fig. 3). Fig. 4 shows the packing diagram of the title compound along the *a* axis.

### S2. Synthesis and crystallization

The title compound was synthesised by adapting a reported procedure (Joseph *et al.*, 2013). A solution of isonicotinic acid hydrazide (0.137 g, 1 mmol) in methanol/DMF (1:1 v/v, 10 ml) was mixed with a methanol /DMF solution (10 ml) of 4-formyl­phenyl acetate (0.164 g, 1 mmol). The mixture was refluxed for 6 h and then cooled to room temperature. The resulting solid was recrystallized from chloro­form/methanol (1:1 v/v). Colorless, block shaped crystals suitable for XRD studies were obtained after slow evaporation of the solution in air over several days.

### S3. Refinement

Crystal data, data collection and structure refinement details are summarized in Table 1. All H atoms bound to C were placed in calculated positions, guided by difference maps, with C—H bond distances of 0.93-0.96 Å. H atoms were assigned *U*_iso_(H) values of 1.2Ueq(carrier). H2 on N2 and H1S & H2S of the water molecule were located in a difference Fourier map and refined with the bond distances restrained to 0.88±0.01 and 0.86±0.02 Å respectively. The low angle reflection (1 0 0) was omitted from the refinement.

### Crystal data

**Table:**

C_15_H_13_N_3_O_3_·H_2_O	*F*(000) = 632
*M**_r_* = 301.30	*D*_x_ = 1.359 Mg m^−^^3^
Monoclinic, *P*2_1_/*c*	Mo *K*α radiation, λ = 0.71073 Å
*a* = 17.3297 (15) Å	Cell parameters from 4335 reflections
*b* = 7.3058 (7) Å	θ = 3.1–28.1°
*c* = 12.4632 (10) Å	µ = 0.10 mm^−^^1^
β = 111.034 (3)°	*T* = 296 K
*V* = 1472.8 (2) Å^3^	Block, colorless
*Z* = 4	0.50 × 0.45 × 0.40 mm

### Data collection

**Table:**

Bruker APEXII CCD diffractometer	2614 independent reflections
Radiation source: fine-focus sealed tube	2153 reflections with *I* > 2σ(*I*)
Graphite monochromator	*R*_int_ = 0.028
ω and φ scans	θ_max_ = 25.1°, θ_min_ = 3.1°
Absorption correction: multi-scan (*SADABS*; Bruker, 2004)	*h* = −20→15
*T*_min_ = 0.951, *T*_max_ = 0.961	*k* = −8→8
8648 measured reflections	*l* = −11→14

### Refinement

**Table:**

Refinement on *F*^2^	Hydrogen site location: mixed
Least-squares matrix: full	H atoms treated by a mixture of independent and constrained refinement
*R*[*F*^2^ > 2σ(*F*^2^)] = 0.037	*w* = 1/[σ^2^(*F*_o_^2^) + (0.0674*P*)^2^ + 0.4633*P*] where *P* = (*F*_o_^2^ + 2*F*_c_^2^)/3
*wR*(*F*^2^) = 0.113	(Δ/σ)_max_ < 0.001
*S* = 0.94	Δρ_max_ = 0.21 e Å^−^^3^
2614 reflections	Δρ_min_ = −0.17 e Å^−^^3^
213 parameters	Extinction correction: *SHELXL2014* (Sheldrick, 2008), Fc^*^=kFc[1+0.001xFc^2^λ^3^/sin(2θ)]^-1/4^
4 restraints	Extinction coefficient: 0.024 (3)

### Special details

**Table:**

Geometry. All e.s.d.'s (except the e.s.d. in the dihedral angle between two l.s. planes) are estimated using the full covariance matrix. The cell e.s.d.'s are taken into account individually in the estimation of e.s.d.'s in distances, angles and torsion angles; correlations between e.s.d.'s in cell parameters are only used when they are defined by crystal symmetry. An approximate (isotropic) treatment of cell e.s.d.'s is used for estimating e.s.d.'s involving l.s. planes.

### Fractional atomic coordinates and isotropic or equivalent isotropic displacement parameters (Å^2^)

**Table:**

	*x*	*y*	*z*	*U*_iso_*/*U*_eq_	
C1	0.16188 (10)	0.0685 (2)	0.93233 (14)	0.0400 (4)	
H1	0.2096	0.0125	0.9817	0.048*	
C2	0.08732 (10)	0.0473 (2)	0.94865 (14)	0.0427 (4)	
H2	0.0868	−0.0231	1.0105	0.051*	
C3	0.01996 (10)	0.2228 (3)	0.79347 (15)	0.0476 (4)	
H3	−0.0287	0.2763	0.7449	0.057*	
C4	0.09113 (10)	0.2527 (2)	0.77045 (14)	0.0423 (4)	
H4	0.0899	0.3245	0.7082	0.051*	
C5	0.16422 (9)	0.1743 (2)	0.84140 (12)	0.0340 (4)	
C6	0.24113 (9)	0.2125 (2)	0.81590 (13)	0.0369 (4)	
C7	0.45140 (9)	0.2232 (2)	0.97566 (13)	0.0367 (4)	
H7	0.4483	0.1754	1.0431	0.044*	
C8	0.53158 (9)	0.2825 (2)	0.97427 (13)	0.0340 (4)	
C9	0.54021 (9)	0.3707 (2)	0.87968 (13)	0.0380 (4)	
H9	0.4941	0.3887	0.8134	0.046*	
C10	0.61707 (10)	0.4314 (2)	0.88411 (13)	0.0388 (4)	
H10	0.6229	0.4915	0.8216	0.047*	
C11	0.68496 (9)	0.4011 (2)	0.98292 (14)	0.0364 (4)	
C12	0.67832 (9)	0.3173 (2)	1.07787 (13)	0.0397 (4)	
H12	0.7247	0.3000	1.1440	0.048*	
C13	0.60111 (10)	0.2590 (2)	1.07295 (14)	0.0395 (4)	
H13	0.5957	0.2030	1.1369	0.047*	
C14	0.80143 (9)	0.3873 (2)	0.92676 (13)	0.0367 (4)	
C15	0.88679 (10)	0.4588 (3)	0.95589 (16)	0.0483 (4)	
H15A	0.9127	0.3993	0.9089	0.072*	
H15B	0.9181	0.4351	1.0354	0.072*	
H15C	0.8847	0.5884	0.9421	0.072*	
N1	0.01652 (8)	0.1220 (2)	0.88093 (12)	0.0455 (4)	
N2	0.31362 (7)	0.18494 (18)	0.90293 (11)	0.0358 (3)	
N3	0.38562 (8)	0.23535 (18)	0.88687 (11)	0.0369 (3)	
O1	0.23649 (7)	0.2689 (2)	0.72122 (10)	0.0589 (4)	
O1S	0.35113 (8)	0.4455 (2)	0.64055 (10)	0.0480 (3)	
O2	0.76382 (7)	0.46558 (16)	0.99337 (10)	0.0446 (3)	
O3	0.76890 (8)	0.27267 (19)	0.85709 (12)	0.0598 (4)	
H1S	0.3278 (13)	0.387 (3)	0.6800 (18)	0.080 (7)*	
H2S	0.3334 (15)	0.554 (2)	0.644 (2)	0.094 (9)*	
H2'	0.3186 (11)	0.148 (2)	0.9725 (10)	0.049 (5)*	

### Atomic displacement parameters (Å^2^)

**Table:**

	*U*^11^	*U*^22^	*U*^33^	*U*^12^	*U*^13^	*U*^23^
C1	0.0310 (8)	0.0445 (10)	0.0443 (9)	−0.0027 (7)	0.0131 (7)	0.0066 (7)
C2	0.0399 (9)	0.0451 (10)	0.0471 (9)	−0.0080 (8)	0.0205 (7)	0.0024 (7)
C3	0.0328 (9)	0.0593 (12)	0.0497 (10)	0.0064 (8)	0.0138 (7)	0.0032 (8)
C4	0.0376 (9)	0.0498 (10)	0.0412 (8)	0.0021 (8)	0.0162 (7)	0.0055 (7)
C5	0.0308 (8)	0.0363 (9)	0.0357 (8)	−0.0045 (7)	0.0132 (6)	−0.0039 (6)
C6	0.0343 (8)	0.0403 (9)	0.0385 (8)	−0.0059 (7)	0.0158 (7)	0.0003 (7)
C7	0.0347 (9)	0.0365 (9)	0.0435 (9)	−0.0015 (7)	0.0199 (7)	0.0004 (7)
C8	0.0303 (8)	0.0330 (8)	0.0419 (8)	0.0002 (6)	0.0169 (6)	−0.0039 (6)
C9	0.0317 (8)	0.0445 (9)	0.0373 (8)	−0.0010 (7)	0.0118 (6)	−0.0013 (7)
C10	0.0391 (9)	0.0441 (9)	0.0379 (8)	−0.0048 (7)	0.0196 (7)	−0.0012 (7)
C11	0.0304 (8)	0.0386 (9)	0.0443 (8)	−0.0071 (7)	0.0184 (7)	−0.0121 (7)
C12	0.0315 (8)	0.0465 (10)	0.0395 (8)	0.0005 (7)	0.0106 (7)	−0.0013 (7)
C13	0.0388 (9)	0.0426 (9)	0.0400 (8)	−0.0003 (7)	0.0176 (7)	0.0040 (7)
C14	0.0351 (8)	0.0394 (9)	0.0378 (8)	0.0001 (7)	0.0157 (7)	−0.0018 (7)
C15	0.0375 (9)	0.0532 (11)	0.0600 (10)	−0.0045 (8)	0.0246 (8)	−0.0013 (8)
N1	0.0352 (8)	0.0526 (9)	0.0534 (8)	−0.0053 (7)	0.0216 (7)	−0.0049 (7)
N2	0.0286 (7)	0.0437 (8)	0.0394 (7)	−0.0049 (6)	0.0174 (6)	0.0019 (6)
N3	0.0305 (7)	0.0402 (8)	0.0455 (7)	−0.0042 (6)	0.0203 (6)	−0.0011 (6)
O1	0.0413 (7)	0.0937 (11)	0.0431 (7)	−0.0115 (7)	0.0168 (5)	0.0149 (7)
O1S	0.0471 (7)	0.0572 (9)	0.0459 (7)	0.0022 (6)	0.0241 (6)	0.0071 (6)
O2	0.0342 (6)	0.0543 (7)	0.0512 (7)	−0.0151 (5)	0.0223 (5)	−0.0191 (5)
O3	0.0482 (8)	0.0701 (9)	0.0660 (8)	−0.0102 (7)	0.0266 (6)	−0.0306 (7)

### Geometric parameters (Å, º)

**Table:**

C1—C2	1.387 (2)	C9—H9	0.9300
C1—C5	1.384 (2)	C10—C11	1.382 (2)
C1—H1	0.9300	C10—H10	0.9300
C2—N1	1.331 (2)	C11—C12	1.374 (2)
C2—H2	0.9300	C11—O2	1.4066 (18)
C3—N1	1.334 (2)	C12—C13	1.384 (2)
C3—C4	1.380 (2)	C12—H12	0.9300
C3—H3	0.9300	C13—H13	0.9300
C4—C5	1.382 (2)	C14—O3	1.1921 (19)
C4—H4	0.9300	C14—O2	1.3527 (18)
C5—C6	1.503 (2)	C14—C15	1.486 (2)
C6—O1	1.2252 (19)	C15—H15A	0.9600
C6—N2	1.348 (2)	C15—H15B	0.9600
C7—N3	1.275 (2)	C15—H15C	0.9600
C7—C8	1.462 (2)	N2—N3	1.3827 (17)
C7—H7	0.9300	N2—H2'	0.883 (9)
C8—C13	1.389 (2)	O1S—H1S	0.857 (16)
C8—C9	1.398 (2)	O1S—H2S	0.860 (16)
C9—C10	1.386 (2)		
			
C2—C1—C5	119.01 (15)	C11—C10—H10	120.6
C2—C1—H1	120.5	C9—C10—H10	120.6
C5—C1—H1	120.5	C12—C11—C10	122.10 (14)
N1—C2—C1	123.81 (15)	C12—C11—O2	116.65 (14)
N1—C2—H2	118.1	C10—C11—O2	121.14 (14)
C1—C2—H2	118.1	C11—C12—C13	118.52 (14)
N1—C3—C4	124.23 (16)	C11—C12—H12	120.7
N1—C3—H3	117.9	C13—C12—H12	120.7
C4—C3—H3	117.9	C12—C13—C8	121.23 (15)
C3—C4—C5	118.91 (15)	C12—C13—H13	119.4
C3—C4—H4	120.5	C8—C13—H13	119.4
C5—C4—H4	120.5	O3—C14—O2	122.55 (14)
C4—C5—C1	117.75 (14)	O3—C14—C15	126.45 (15)
C4—C5—C6	117.81 (14)	O2—C14—C15	110.97 (14)
C1—C5—C6	124.43 (14)	C14—C15—H15A	109.5
O1—C6—N2	123.04 (14)	C14—C15—H15B	109.5
O1—C6—C5	120.63 (14)	H15A—C15—H15B	109.5
N2—C6—C5	116.31 (13)	C14—C15—H15C	109.5
N3—C7—C8	121.78 (14)	H15A—C15—H15C	109.5
N3—C7—H7	119.1	H15B—C15—H15C	109.5
C8—C7—H7	119.1	C2—N1—C3	116.29 (14)
C13—C8—C9	118.84 (14)	C6—N2—N3	118.20 (12)
C13—C8—C7	118.62 (14)	C6—N2—H2'	124.8 (11)
C9—C8—C7	122.45 (14)	N3—N2—H2'	116.7 (11)
C10—C9—C8	120.40 (14)	C7—N3—N2	115.33 (13)
C10—C9—H9	119.8	H1S—O1S—H2S	100.4 (19)
C8—C9—H9	119.8	C14—O2—C11	117.88 (12)
C11—C10—C9	118.88 (14)		
			
C5—C1—C2—N1	0.6 (3)	C9—C10—C11—O2	−177.75 (14)
N1—C3—C4—C5	0.1 (3)	C10—C11—C12—C13	1.1 (2)
C3—C4—C5—C1	0.2 (2)	O2—C11—C12—C13	177.22 (14)
C3—C4—C5—C6	−178.71 (15)	C11—C12—C13—C8	0.6 (2)
C2—C1—C5—C4	−0.5 (2)	C9—C8—C13—C12	−1.5 (2)
C2—C1—C5—C6	178.31 (14)	C7—C8—C13—C12	−178.10 (15)
C4—C5—C6—O1	−18.5 (2)	C1—C2—N1—C3	−0.3 (2)
C1—C5—C6—O1	162.62 (17)	C4—C3—N1—C2	−0.1 (3)
C4—C5—C6—N2	160.04 (15)	O1—C6—N2—N3	4.8 (2)
C1—C5—C6—N2	−18.8 (2)	C5—C6—N2—N3	−173.74 (12)
N3—C7—C8—C13	−177.64 (15)	C8—C7—N3—N2	−176.06 (13)
N3—C7—C8—C9	5.9 (2)	C6—N2—N3—C7	173.26 (14)
C13—C8—C9—C10	0.8 (2)	O3—C14—O2—C11	3.2 (2)
C7—C8—C9—C10	177.24 (15)	C15—C14—O2—C11	−174.82 (14)
C8—C9—C10—C11	0.8 (2)	C12—C11—O2—C14	115.34 (16)
C9—C10—C11—C12	−1.8 (2)	C10—C11—O2—C14	−68.5 (2)

### Hydrogen-bond geometry (Å, º)

**Table:**

*D*—H···*A*	*D*—H	H···*A*	*D*···*A*	*D*—H···*A*
C1—H1···O1*S*^i^	0.93	2.56	3.375 (2)	147
C7—H7···O1*S*^i^	0.93	2.56	3.3655 (19)	145
C12—H12···O3^i^	0.93	2.54	3.329 (2)	143
N2—H2′···O1*S*^i^	0.88 (1)	2.08 (1)	2.9529 (18)	170 (2)
O1*S*—H1*S*···N3	0.86 (2)	2.65 (2)	3.2897 (18)	133 (2)
O1*S*—H1*S*···O1	0.86 (2)	2.02 (2)	2.8382 (17)	159 (2)
O1*S*—H2*S*···O3^ii^	0.86 (2)	2.38 (2)	3.1754 (19)	154 (2)

Symmetry codes: (i) *x*, −*y*+1/2, *z*+1/2; (ii) −*x*+1, *y*+1/2, −*z*+3/2.
