# Supplementary figures and images for: Crystal structure of 4-{(E)-[2-(pyridin-4-ylcarbon­yl)hydrazin-1-yl­idene]meth­yl}phenyl acetate monohydrate
Source: Acta Crystallogr E Crystallogr Commun. 2015 Jan 3;71(Pt 2):o79–80. doi: 10.1107/S2056989014027819 (PMC4384571; doi:10.1107/S2056989014027819)

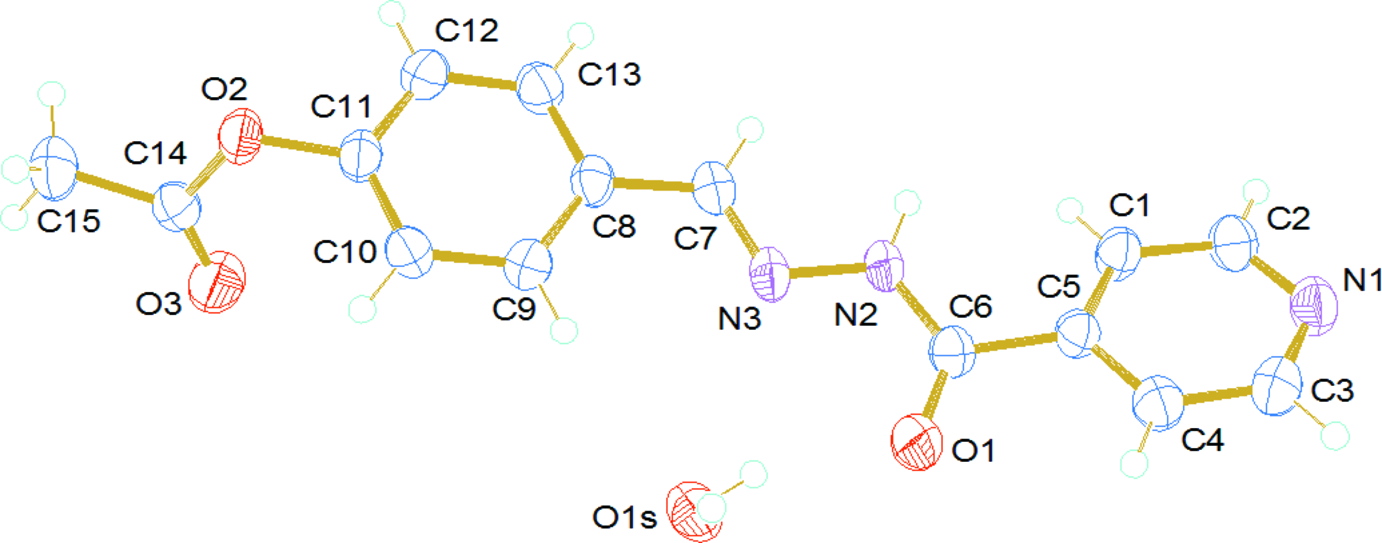

Supplement: Supplementary file 4 [file e-71-00o79-fig1.tif]

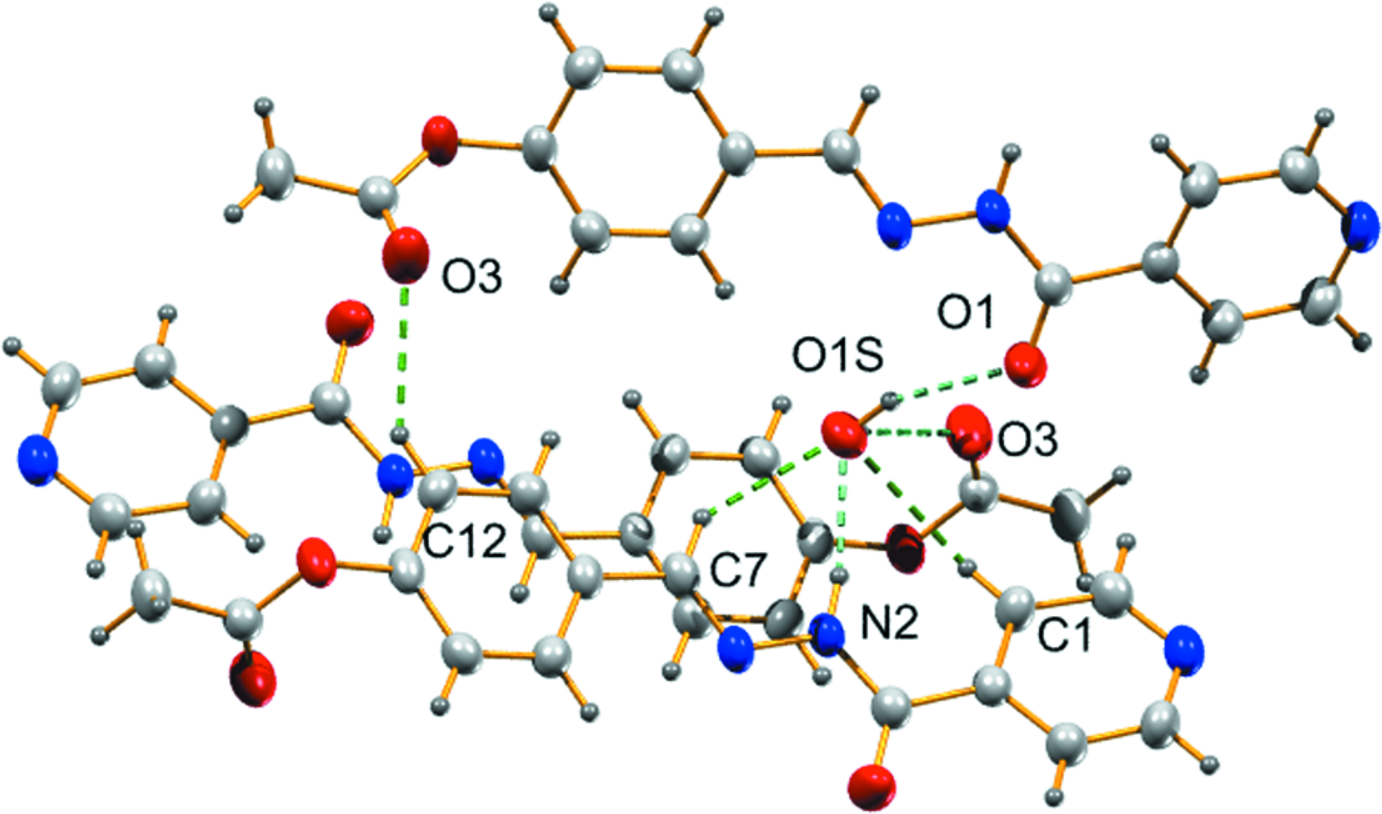

Supplement: Supplementary file 5 [file e-71-00o79-fig2.tif]

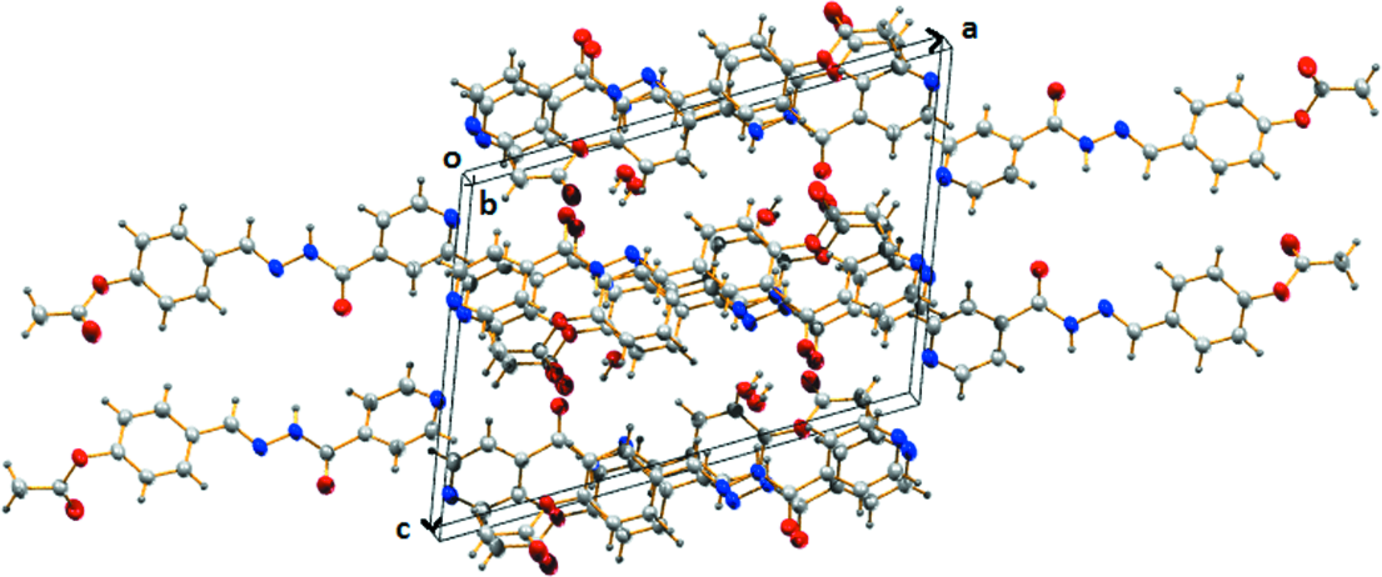

Supplement: Supplementary file 6 [file e-71-00o79-fig3.tif]

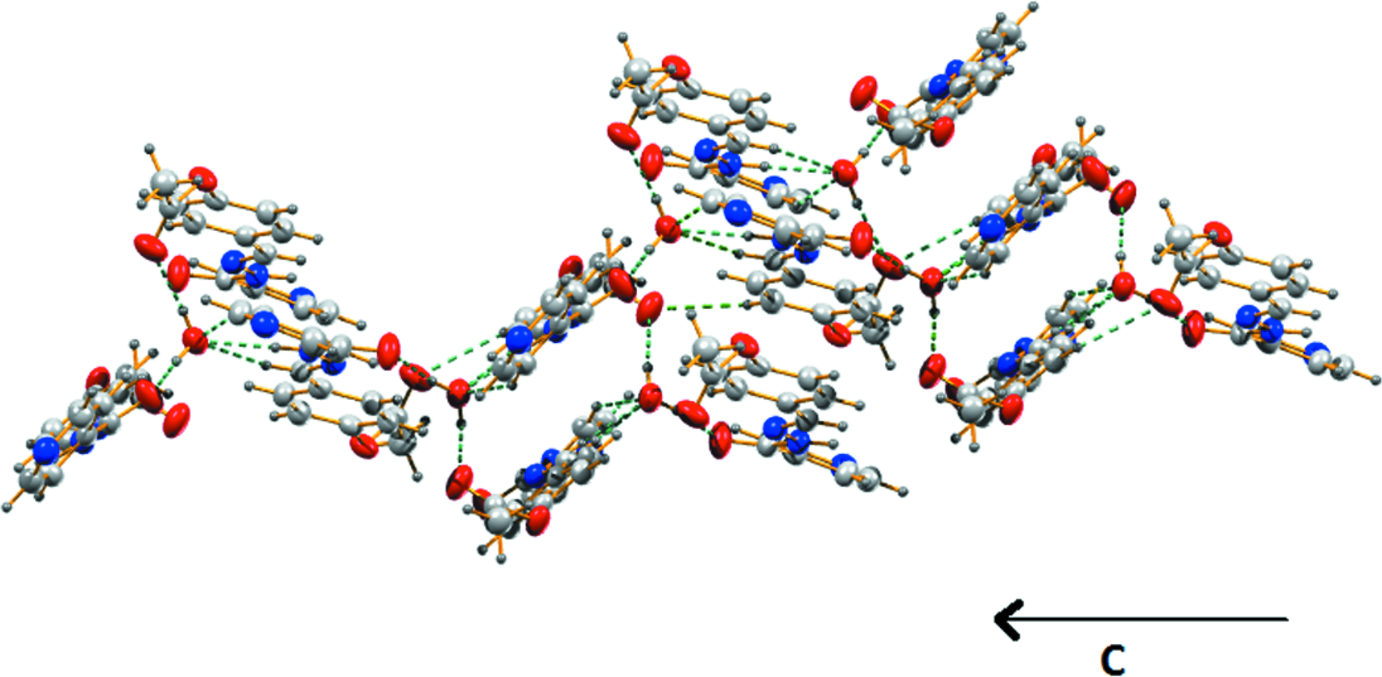

Supplement: Supplementary file 7 [file e-71-00o79-fig4.tif]
